# Effects of eight weeks of functional training with blood flow restriction on biochemical, cognitive, and functional outcomes in postmenopausal women: protocol for a single-blind randomized controlled trial

**DOI:** 10.3389/fspor.2026.1953943

**Published:** 2026-10-27

**Authors:** Elham Shakoor, Faezeh Aliakbari Jam, Mostafa Khafaei, Lara Carneiro, Sadegh Amani-Shalamzari

**Affiliations:** 1 Department of Exercise Physiology, Faculty of Sport Sciences, Alzahra University, Tehran, Iran; 2 Human Genetics Research Center, Baqiyatallah Medical Science University, Tehran, Iran; 3 Physical Education Department, College of Education, United Arab Emirates University, Al Ain, Abu Dhabi, United Arab Emirates; 4 Department of Exercise Physiology, Faculty of Sports Science, Kharazmi University, Tehran, Iran

**Keywords:** blood flow restriction training, cognition, muscle strength, postmenopausal, women

## Abstract

**Introduction:**

Aging in women is accompanied by impaired muscle and cognitive function, reduced anabolic activity, and chronic low-grade inflammation. Blood flow restriction (BFR) may enhance adaptations to low-load exercise, but its effects when combined with functional training in postmenopausal women are unclear. This trial will examine the effects of eight weeks of functional training with BFR on biochemical, cognitive, and functional outcomes in postmenopausal women.

**Methods:**

This protocol describes a single-blind, assessor-masked, randomized controlled trial that will recruit 45 eligible postmenopausal women, defined as having experienced no menstrual bleeding for at least 24 consecutive months before screening, without another identifiable physiological or pathological cause. After health screening and physician-confirmed examination, participants will be randomly assigned (1:1:1) to functional training with BFR (FTBFR; *n* = 15), functional training alone (FT; *n* = 15), or non-exercise control (CON; *n* = 15) using web-based randomization. Exercise groups will complete supervised sessions three times weekly for eight weeks. Primary outcomes are plasma/serum concentrations of growth hormone, insulin-like growth factor-binding protein-3, interleukin-6, C-reactive protein, vascular endothelial growth factor, testosterone, and cortisol, measured by ELISA; the testosterone-to-cortisol ratio will also be calculated. Cognitive outcomes include the Mini-Mental State Examination, Montreal Cognitive Assessment, Stroop test, and Corsi block-tapping test. Secondary outcomes are handgrip strength, upper- and lower-body muscle strength, Timed Up and Go five-times-sit-to-stand performance, quality of life (SF-36), and pain.

**Conclusions:**

This trial will clarify whether adding BFR to functional training provides additional biochemical, cognitive, and functional benefits in Postmenopausal women and may inform safe, low-intensity exercise strategies for healthy aging.

**Clinical Trial Registration:**

https://en.irct.ir/trial/89951, IRCT20191123045478N4.

## 1 Introduction

Menopause is a major biological transition that may accelerate age-related declines in musculoskeletal, metabolic, vascular, and cognitive health. Although chronological aging contributes to these changes, the menopausal transition introduces additional physiological stressors, particularly the sustained reduction in ovarian estrogen production. With the continuing growth of the global population of older women, identifying safe and effective strategies to preserve physical function, cognitive health, and independence in postmenopausal women has become an important public health priority (1, 2).

One of the major clinical consequences of aging and menopause is sarcopenia, which is characterized by progressive reductions in muscle strength, muscle mass, and physical performance. In postmenopausal women, estrogen deficiency may exacerbate this process by reducing muscle protein turnover, impairing neuromuscular function, increasing adipose tissue accumulation, and intensifying systemic inflammation (3, 4). Estrogen receptors are expressed in skeletal muscle and may influence satellite-cell activity, mitochondrial function, muscle repair, and the regulation of inflammatory responses. Consequently, the postmenopausal decline in estrogen may reduce the capacity of skeletal muscle to adapt to physical stress and increase vulnerability to muscle weakness, mobility limitations, falls, and loss of functional independence (4, 5).

The pathophysiology underlying muscle loss in postmenopausal women involves complex interactions among hormonal alterations, anabolic resistance, inflammation, and neuromuscular dysfunction. The growth hormone (GH)/insulin-like growth factor-1 (IGF-1) axis, together with insulin-like growth factor-binding protein-3 (IGFBP-3), plays an important role in muscle protein synthesis and tissue repair through the PI3 K/Akt/mTOR pathway. Aging and estrogen deficiency may impair anabolic signaling and reduce the responsiveness of skeletal muscle to nutritional and exercise stimuli. Consequently, physiological balance may shift toward proteolytic pathways, including the ubiquitin–proteasome system and the autophagy–lysosome pathway (5–7). The testosterone-to-cortisol ratio (TCR) may provide a composite, although indirect, indicator of the balance between anabolic and catabolic endocrine activity. A lower TCR has been associated with a more catabolic physiological environment, impaired muscle maintenance, greater adiposity, and poorer neuromuscular function (8–10). Testosterone may support muscle repair and remodeling through androgen-receptor signaling and interactions with the IGF-1 pathway, whereas cortisol stimulates proteolysis and, when chronically elevated, may disrupt insulin- and IGF-1-dependent anabolic signaling (8–10).

Concurrently, chronic low-grade inflammation may further inhibit anabolic signaling and contribute to muscle dysfunction in postmenopausal women. Inflammatory mediators, including interleukin-6 (IL-6), C-reactive protein (CRP), and tumor necrosis factor-alpha (TNF-α), may interfere with GH/IGF-1 signaling and contribute to anabolic resistance. Specifically, IL-6 may increase hepatic CRP production through the JAK/STAT3 pathway and suppress GH-related signaling by inducing suppressor proteins involved in cytokine signaling. These mechanisms may reduce the bioavailability of IGF-1 and IGFBP-3, thereby impairing muscle protein synthesis and recovery processes (7, 8, 11). Thus, the combined effects of estrogen deficiency, inflammation, and diminished anabolic responsiveness may place postmenopausal women at increased risk of sarcopenia and functional decline.

As part of this pathophysiological process, chronic low-grade inflammation may extend beyond skeletal muscle and adversely affect neurovascular and cognitive health in postmenopausal women. Estrogen contributes to the regulation of cerebral blood flow, endothelial function, neurotransmitter activity, mitochondrial function, synaptic plasticity, and neuroprotective mechanisms. Therefore, the sustained reduction in estrogen during and after the menopausal transition may increase the vulnerability of the brain to the adverse effects of inflammation and vascular dysfunction. Under these conditions, elevated inflammatory mediators, particularly IL-6 and CRP, may impair brain regions involved in memory and executive function, including the hippocampus and prefrontal cortex, through endothelial dysfunction, disruption of blood–brain barrier integrity, microglial activation, and impaired neurogenesis and synaptic plasticity (12–14). Accordingly, the same inflammatory environment that contributes to muscle dysfunction through GH/IGF-1 resistance and impaired protein synthesis may simultaneously promote cognitive decline and reduced cognitive–motor coordination by disrupting neural processes.

In this context, vascular endothelial growth factor (VEGF) may represent an important link between vascular health, neural function, and muscular adaptation. In addition to its central role in angiogenesis, VEGF contributes to the regulation of cerebral blood flow, neurogenesis, and the trophic support of neurons. However, aging and reduced estrogenic support may be accompanied by diminished activity of VEGF-related pathways, potentially limiting the ability of cerebral vessels to meet the metabolic demands of neural tissue and maintain adequate cerebral perfusion. Available evidence suggests that age-related reductions in VEGF, particularly in the presence of chronic inflammation, may be associated with impaired cerebrovascular reactivity and poorer outcomes in postmenopausal women (14, 15). Moreover, reduced cerebral blood flow and cerebrovascular dysfunction, in interaction with systemic inflammation and estrogen deficiency, may exacerbate declines in executive function, processing speed, and memory and increase the risk of mild cognitive impairment and dementia (14, 16).

High-intensity resistance training is effective for improving muscle strength and physical performance; however, it may not be well tolerated by all postmenopausal women, particularly those with joint pain, low baseline strength, mobility limitations, or fear of injury. Accordingly, low-load and population-specific exercise strategies may provide a practical alternative. Blood flow restriction (BFR) exercise combines low-load exercise with partial restriction of limb blood flow and may activate anabolic and angiogenic pathways, including the PI3 K/Akt/mTOR pathway and VEGF-related signaling, through localized metabolic stress and hypoxic stimuli (17, 18). These responses may support improvements in muscle strength and physical function while reducing the external loads required during exercise. Nevertheless, cuff pressure, exercise intensity, training frequency, and participant characteristics may influence the physiological response and safety profile of BFR.

Despite increasing interest in BFR, evidence specific to postmenopausal women remains limited. Women, particularly middle-aged and older women, have been underrepresented in resistance-training studies, which may limit the generalizability of existing findings to postmenopausal women and overlook the generalizability of existing findings to postmenopausal women and overlook sex (19) Although a systematic review and meta-analysis suggested that BFR may improve selected indices of muscle strength, physical performance, and cardiovascular health in middle-aged and older women, the limited number of studies and considerable heterogeneity among exercise protocols remain important limitations (20). Therefore, investigating the efficacy and safety of BFR specifically in postmenopausal women is necessary to clarify its clinical applicability and inform the development of exercise interventions tailored to this population.

In this regard, an approach that combines the potential benefits of BFR with the transfer of muscular and circulatory adaptations to purposeful movements and activities of daily living warrants consideration. Functional training (FT) may provide such a framework. FT emphasizes multijoint and activity-specific movement patterns that resemble everyday tasks, including sit-to-stand transfers, stair negotiation, walking, and dynamic balance (21). This approach may be particularly relevant for postmenopausal women because improvements in isolated muscle strength do not necessarily translate into enhanced mobility or independence unless that strength is applied within functional movement patterns. Functional training may also provide cognitive–motor stimulation when exercises require attention, movement sequencing, postural control, or simultaneous dual-task processing. Previous studies have shown that functional and dual-task exercise can improve physical performance and selected cognitive outcomes in older women, whereas BFR may enhance muscle quality and circulatory adaptations when relatively low external loads are used (17, 18, 20, 21). In a study of older women, dual-task exercise combined with BFR improved muscle quality, body composition, and cognitive–functional performance, supporting the potential value of integrating BFR with activity-specific exercise (22).

Nevertheless, the effects of combining FT with BFR on the interconnected endocrine, inflammatory, angiogenic, cognitive, and functional pathways relevant to postmenopausal women remain insufficiently understood. In particular, evidence regarding the simultaneous effects of this intervention on GH, IGFBP-3, IL-6, CRP, VEGF, TCR, cognitive function, upper- and lower-body strength, mobility, pain, and quality of life gap may clarify whether FT combined gap may clarify whether FT combined with BFR represents a feasible and clinically relevant strategy for mitigating the consequences of estrogen deficiency, inflammation, muscle weakness, and cognitive–motor decline in postmenopausal women.

Therefore, this study aimed to investigate the effects of 8 weeks of functional training combined with blood flow restriction on serum GH, IGFBP-3, TCR, cognitive function, upper- and lower-body muscle strength, functional performance, quality of life, and pain in postmenopausal women. We hypothesized that, compared with functional training alone and a non-exercising control condition, 8 weeks of functional training combined with BFR would increase GH, IGFBP-3, VEGF, and TCR; decrease IL-6 and CRP; and improve cognitive function, upper- and lower-body muscle strength, functional performance, quality of life, and pain in postmenopausal women.

## 2 Article types

Study Protocol

## 3 Materials and methods

### 3.1 Study design

This study is designed as a single-blind, three-arm, parallel-group randomized controlled trial protocol to investigate the effects of eight weeks of functional training with blood flow restriction on biochemical, cognitive, and functional outcomes in Postmenopausal women. Eligible participants will be randomly assigned to one of the following three groups:

1. Functional training with blood flow restriction (FTBFR)
2. Functional training without blood flow restriction (FT)
3. Control group (CON)

Outcome assessors and laboratory personnel will be blinded to group allocation.

This protocol has been developed in accordance with the SPIRIT guidelines. The final trial report will be prepared in accordance with the CONSORT recommendations for randomized controlled trials, and the completed CONSORT checklist is provided as Supplementary File 1 (23, 24). Table 1 presents the schedule of participant enrolment, group allocation, intervention sessions, and outcome assessments.

**Table 1:** SPIRIT Checklist for schedule of enrolment, interventions, and assessments.

Trial period					
Study parameter	Enrollment	Allocation	Baseline	After 8 weeks	
Timepoint^b^	*-t_I_*	0	Pr₁	Po₁	Po2
Enrollment:					
Eligibility screen	X				
Informed consent form	X				
socio-demographic and clinical profile	X				
Randomization		X			
Allocation		X			
Intervention/ comparator:					
FTBFR		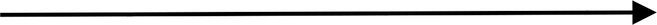			
FT		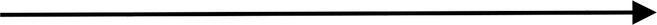			
CON		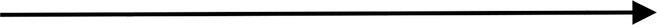			
Assessments:					
Food Frequency Questionnaire (FFQ)					
General Health Questionnaire (GHQ)	X				
BMI (Body Mass Index)	X				X
Serum Levels of GH, IGFBP-3, IL-6,CRP, VEGF, testosterone/cortisol ratio,	X				X
cognitive function (MMSE, MoCA, Stroop test and Corsi block-tapping test)	X				X
Muscle Strength (Bench Press, Leg Press)	X				X
Functional Performance (TUG, 5×STS)	X				X
Quality of Life (SF-36)	X			X	
Pain Intensity (VAS)	X			X	

^b^Study time points and schedule of assessments. FTBFR, functional training with blood flow restriction group; FT, functional training group; CON, Control group; -t₁, pre pre-allocation time point; Pr1, Pre-test; Po1, Post-test; Po2, Post-test (48 h After 8 Weeks); GH, growth hormone; IGFBP-3, insulin-like growth factor binding protein 3; IL-6, interleukin-6; CR,: C-Reactive Protein; VEGF, Vascular Endothelial Growth Factor; MMSE, Mini-Mental State Examination; MoCA, Montreal Cognitive Assessment; TUG, timed up-and-go test; 5×STS, Five-Repetition Sit-to-Stand test; SF-36, Short Form 36; VAS, Visual Analogue Scale**.**

### 3.2 Trial registration and ethics

The study protocol has been reviewed and approved by the Research Ethics Committee of Kharazmi University (IR.KHU.REC.1405.020; 13 April 2026) and will be conducted in accordance with the principles of the Declaration of Helsinki. The trial has also been registered in the Iranian Registry of Clinical Trials (IRCT20191123045478N4; 25 May 2026). Written informed consent was obtained from all participants prior to study enrolment.

Participant recruitment is planned to commence on 15 June 2026 and is expected to continue until 21 August 2026. Outcome assessments will be conducted at baseline and 48 h after completion of the 8-week intervention period.

### 3.3 Participants

#### 3.3.1 Eligibility criteria

Community-dwelling Postmenopausal women will be recruited through local advertisements and public announcements. Individuals meeting the eligibility criteria outlined below will be considered for inclusion in the trial.

##### 3.3.1.1 Inclusion criteria

Participants will be eligible if they meet all of the following criteria: (1) being a postmenopausal woman, defined as having experienced no menstrual bleeding for at least 24 consecutive months before screening, without another identifiable physiological or pathological cause; (2) ability to participate in supervised exercise sessions; (3) a sedentary or low-active lifestyle, defined as < 150 min/week of moderate-intensity physical activity or < 75 min/week of vigorous-intensity physical activity during the previous 6 months, as assessed using the International Physical Activity Questionnaire–Short Form (IPAQ-SF) (25); (4) no history of smoking or use of any tobacco- or nicotine-containing products, including electronic cigarettes, within the past 5 years; (5) no diagnosed neurological disorders (e.g., Parkinson's disease, multiple sclerosis, or stroke with residual motor impairment), primary myopathies, or severe musculoskeletal disorders, including severe osteoporosis with a history of fracture, rheumatoid arthritis, or knee/hip osteoarthritis associated with pain ≥ 4/10 on the visual analogue scale (VAS) during activities of daily living (26); (6) a score of ≥ 24 on the Mini-Mental State Examination (MMSE), indicating the absence of overt cognitive impairment (27); (7) medical clearance for participation in low- to moderate-intensity exercise; (8) no skin disease, active infection, open wound, or lymphedema in the upper or lower limbs that would contraindicate the application of BFR cuffs; (9) no participation in any regular resistance or functional training programme within the previous 6 months; and (10) provision of written informed consent.

##### 3.3.1.2 Exclusion criteria

Participants will be excluded if they present with any of the following: (1) uncontrolled cardiovascular or metabolic disease precluding safe participation in exercise; (2) a history of thromboembolic disease, severe peripheral vascular disease, or any other medical condition contraindicating blood flow restriction; (3) hypertension, whether controlled or uncontrolled, irrespective of antihypertensive treatment status, or unstable cardiac symptoms; (4) visual or vestibular impairments that preclude safe participation in the exercise intervention or functional assessments; (5) use of medications or supplements likely to substantially influence anabolic, inflammatory, or vascular biomarkers, unless such use is stable and appropriately documented; (6) concurrent participation in another interventional study during the trial period; or (7) inability to attend the intervention sessions regularly.

Following specialist medical examination and confirmation of eligibility, participants will be enrolled in the study. Participants will also be withdrawn from the trial if they develop adverse symptoms during the study, are advised by a physician to discontinue exercise, or no longer meet the predefined safety criteria during the intervention period.

### 3.4 Randomization and allocation concealment

Following completion of baseline assessments, eligible participants will be randomly allocated in a 1:1:1 ratio to one of three parallel groups using a web-based randomization tool **(**randomizer.org**)**: (1) FTBFR, functional training with blood flow restriction; (2) FT, functional training alone; or (3) CON, control. Allocation concealment will be ensured using sequentially numbered, opaque, sealed envelopes prepared by a researcher not involved in either outcome assessment or intervention delivery. Once baseline assessment has been completed and eligibility confirmed, the study coordinator will open the next envelope in sequence to determine the participant's group assignment.

### 3.5 Blinding

This trial will adopt a single-blind design. Owing to the nature of the exercise intervention, blinding of participants and exercise trainers will not be feasible. However, outcome assessors, laboratory personnel, and, where possible, the statistician responsible for the primary analyses will remain blinded to group allocation.

### 3.6 Sample size

The sample size was calculated *a priori* using G*Power version 3.1.9.6 (Heinrich Heine University Düsseldorf, Düsseldorf, Germany) (28). The calculation was based on a repeated-measures ANCOVA framework with three groups (FTBFR, FT, and CON) and two assessment points (baseline and post-intervention). With a two-sided *α* of 0.05, power of 80%, an assumed medium effect size of f = 0.25 (29, 30), and a within-subject correlation of 0.70, the minimum required sample size was 36 participants (12 per group). Allowing for an anticipated attrition rate of approximately 15%, the target enrolment was increased to 45 participants (15 per group). Participant recruitment, allocation, and follow-up will be presented in the trial flow diagram (Figure 1).

**Figure 1:**
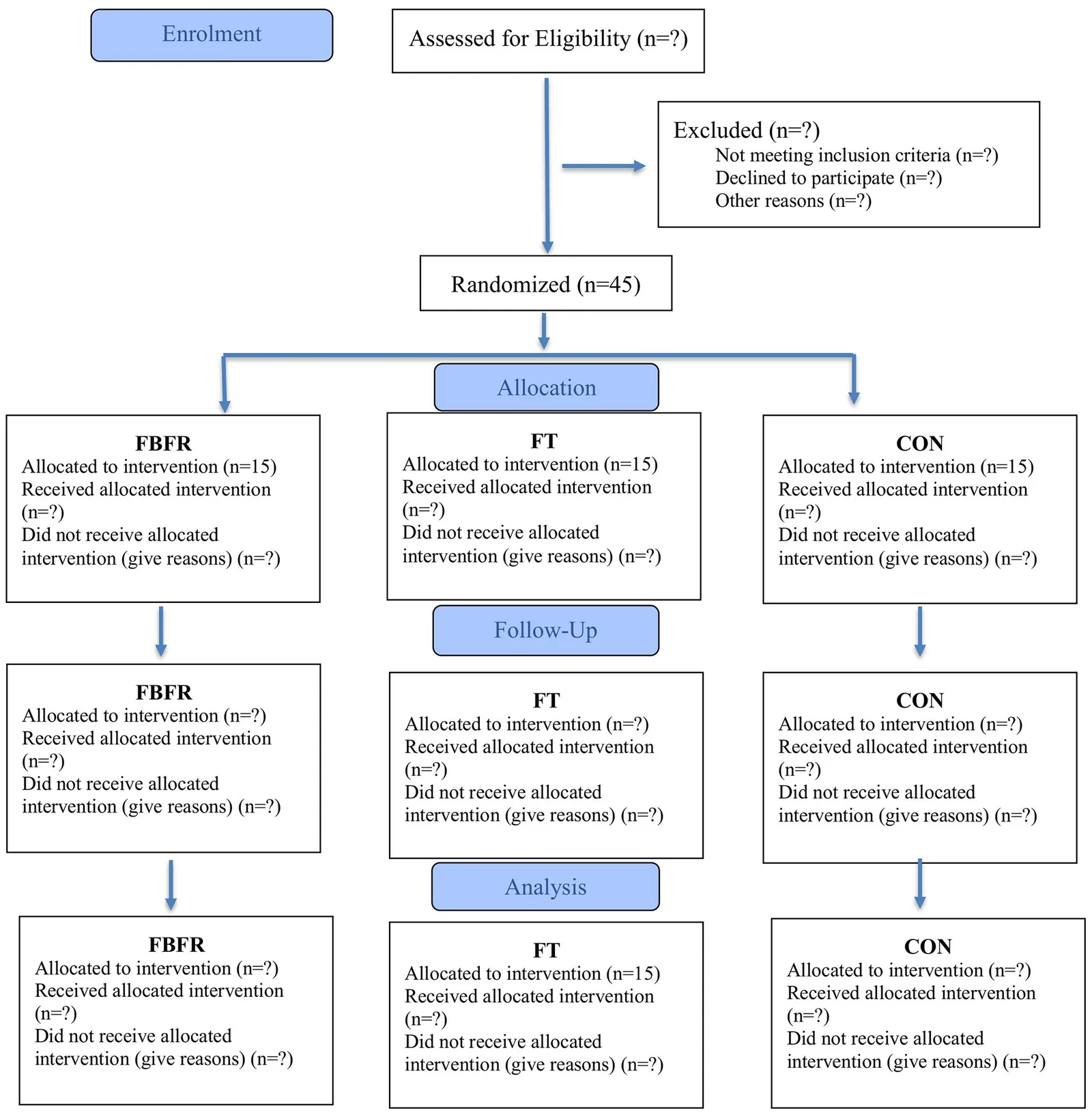
Participant flow chart.

### 3.7 Procedure and interventions

#### 3.7.1 Study procedure

One week prior to commencement, candidates will attend an information session covering study procedures, potential risks, and the right to withdraw. Baseline demographic and clinical characteristics will be recorded by trained assessors, including age, body mass (kg), stature (cm), body mass index (BMI; kg/m2), physical activity level (IPAQ-SF), waist-to-hip ratio, blood pressure, and current medication use.

#### 3.7.2 Interventions

Participants in the two intervention groups will perform an 8-week supervised functional training program, three times per week on non-consecutive days. Each session comprises a 10-minute warm-up (brisk walking and dynamic stretching), a 40–50 min main functional circuit, and a 5–10 min cool-down (static stretching). The intervention follows a circuit-based functional resistance training model, incorporating upper and lower limb resistance, core stability tasks, and balance challenges on stable and unstable surfaces (e.g., BOSU® balls).

##### 3.7.2.1 Functional training protocol (FTBFR and FT groups)

The circuit consists of eight exercises: (1) dumbbell fly on a Swiss ball, (2) medicine ball squat on a BOSU ball, (3) standing shoulder press on a BOSU ball, (4) medicine ball crunches, (5) triceps extension on a Swiss ball, (6) forward lunge on a BOSU ball, (7) biceps curl on a BOSU ball, and (8) medicine ball hypererextension (Figure 2).

**Figure 2:**
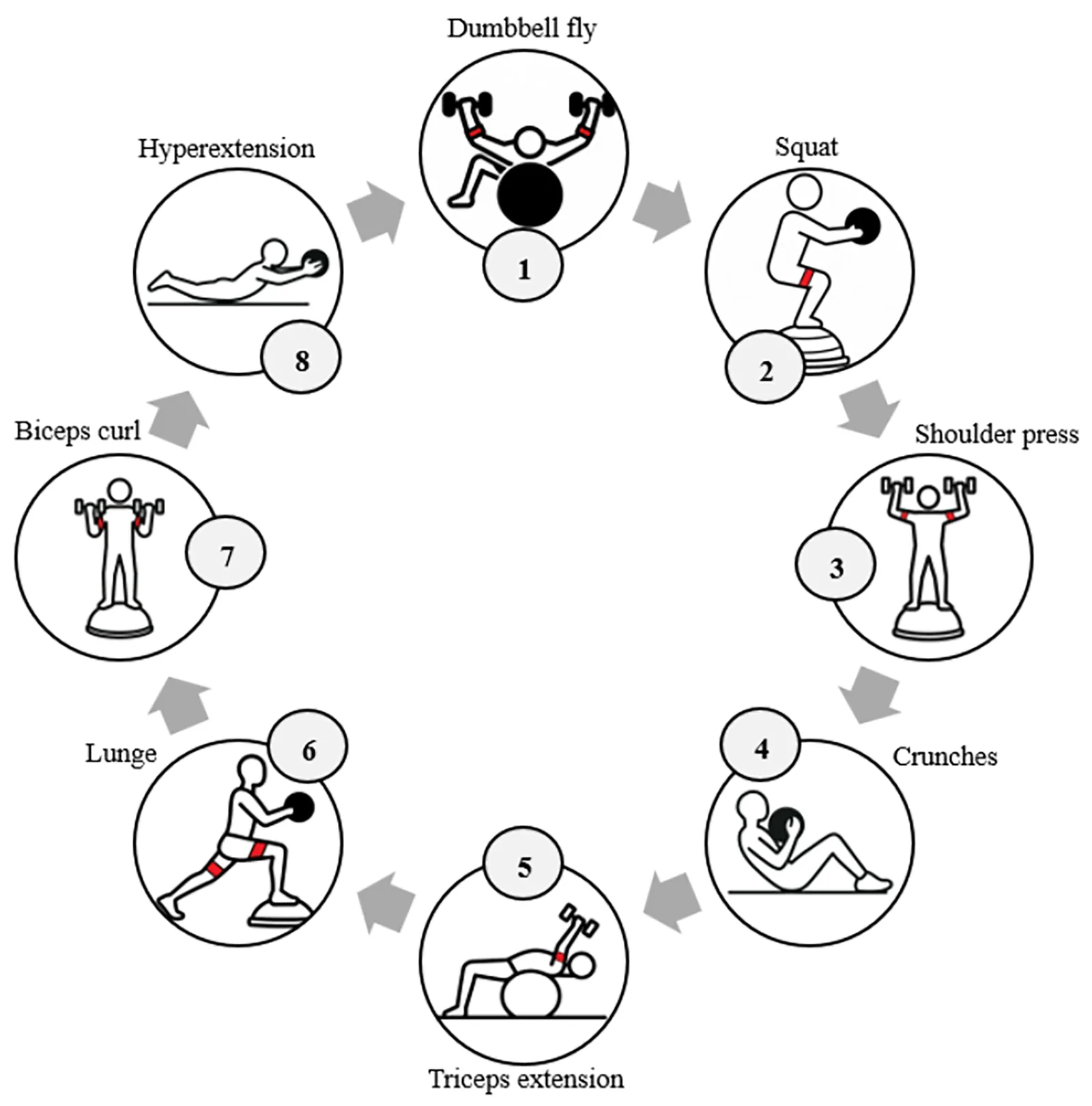
Functional circuit training exercises.

For stations 1, 3, 5, and 7, intensity is prescribed as a percentage of the estimated one-repetition maximum (1RM), calculated via the Brzycki formula (Equation 1) (31). For stations 2, 4, 6, and 8, intensity is based on medicine ball weight (2–5 kg, individualized). Training variables are detailed in Table 2. Briefly, participants perform 2–3 sets of 10 repetitions per station, with 1-minute inter-station rest. Intensity initiates at 20% 1RM, increasing by 5% 1RM weekly. The Borg Rating of Perceived Exertion (RPE 6–20) will be maintained between 13 and 15 (“somewhat hard”).

$$\frac{Weight\;(kg)}{(Reps\times 0.0278)-1.0278}=1RM$$ (1)

**Table 2:** Weekly progression of functional training variables and blood flow restriction (BFR) parameters during the 8-week intervention.

Variables	Intensity			Sets (N)	Repitations (N)	Rest between sets (min)	Target RPE
	Cuff pressure (AOP%)	Dumbblles (%1RM)	Medicine Ball				
Week 1	50% AOP	20% 1RM	2	2	10	1 min	8-9
Week 2	55% AOP	25% 1RM					
Week3	60% AOP	30% 1RM	3	3	10	1 min	10-11
Week 4	65% AOP	35% 1RM					
Week 5	70% AOP	40% 1RM	4	3	10	1 min	12
Week 6	75% AOP	45% 1RM					
Week 7	80% AOP	50% 1RM	5	3	10	1 min	13-14
Week 8	85% AOP	55% 1RM					

Rest intervals between sets and exercises will be standardized according to participant tolerance and safety considerations. Throughout all sessions, exercise technique, breathing pattern, perceived exertion, and the occurrence of any adverse symptoms will be monitored. The primary emphasis of the programme will be on controlled execution of movements rather than lifting maximal loads. Participants will be instructed to avoid breath-holding and the Valsalva manoeuvre, particularly during resistance exercises and under blood flow restriction conditions.

##### 3.7.2.2 Blood flow restriction (BFR) protocol (FTBFR group only)

The FTBFR group will perform the identical exercise protocol with the addition of intermittent BFR. Pneumatic cuffs (Ghamat Pouyan, Iran; width: 5 cm for upper limbs, 8 cm for lower limbs) will be placed proximally on the active limbs.

Arterial Occlusion Pressure (AOP) will be estimated session-specifically using validated anthropometric-based formulas involving limb circumference and blood pressure (Equations 2 and 3) (32, 33). Restriction pressure will progress from 50% AOP in week 1 to 85% AOP by week 8 (Table 2). In the FTBFR group, cuffs will remain inflated at the prescribed pressure throughout the two to three sets of each exercise, including the 60-second inter-set rest intervals. After completion of all sets of an exercise, the cuffs will be fully deflated during the 1–2-minute rest period before transitioning to the next exercise (27).

$$\begin{array}{rl} & \mathrm{Upper}\;\mathrm{body}\;\mathrm{arterial}\;\mathrm{occlusion}\;(\mathrm{mmHg})\;=\;(1.461\times \;\mathrm{arm}\;\mathrm{circumference})\\ & \;+\;(0.514\times \;\mathrm{systolic}\;\mathrm{blood}\;\mathrm{pressure})\;+\\ & \;(0.339\times \;\mathrm{diastolic}\;\mathrm{blood}\;\mathrm{pressure})\;+\;17.236 \end{array}$$ (2)

$$\begin{array}{rl} & \mathrm{Lower}\;\mathrm{body}\;\mathrm{arterial}\;\mathrm{occlusion}\;(\mathrm{mmHg})\;=\;(5.893\times \mathrm{thigh}\;\mathrm{circumference})\\ & \;+\;(0.912\times \;\mathrm{systolic}\;\mathrm{blood}\;\mathrm{pressure})\;+\\ & \;(0.734\times \;\mathrm{diastolic}\;\mathrm{blood}\;\mathrm{pressure})\;-\;220.046 \end{array}$$ (3)

Throughout the training sessions, participants will be continuously monitored for severe pain, numbness, dizziness, unusual shortness of breath, an exaggerated blood pressure response, or any other adverse symptoms. If any of these signs occur, the cuff will be immediately deflated, and the training session will be discontinued or modified as appropriate.

##### 3.7.2.3 Control group

The CON group will be instructed to maintain their habitual lifestyle and avoid initiating any new structured exercise. They will receive general health advice (usual care). Physical activity levels will be monitored to ensure no significant lifestyle changes occur during the study period.

### 3.8 Concomitant care and adverse events

Participants must maintain usual dietary patterns, monitored via a Food Frequency Questionnaire (FFQ**)**. A study physician will be present during FTBFR sessions. Any adverse events (e.g., excessive bruising, persistent numbness, or signs of deep vein thrombosis) will be documented in weekly safety reports. In case of BFR-related distress, cuffs will be immediately deflated and the participant evaluated by the physician.

### 3.9 Participant retention and feedback

To enhance participant retention and adherence, intervention sessions will be scheduled according to participant availability. Participants will be encouraged to provide feedback throughout the intervention regarding exercise intensity, volume, and type. Any concerns or suggestions will be communicated to the research team to facilitate individualized support and, where necessary, minor adjustments to the exercise prescription within the boundaries of the standardized protocol. Participant attendance and the reasons for missed sessions will be recorded systematically.

### 3.10 Outcomes

Assessments will be conducted at baseline and 48 h post-intervention.

#### 3.10.1 Primary outcomes: biochemical markers

Primary outcomes will include the biochemical markers GH, IGFBP-3, IL-6, CRP, VEGF, and the testosterone-to-cortisol ratio (TCR), which reflect anabolic, inflammatory, vascular, and catabolic/anabolic responses. Fasting venous blood samples will be collected from each participant at two time points: before the intervention and 48 h after completion of the intervention.

##### 3.10.1.1 Blood sampling and biochemical marker assessment

Venous blood samples (8 mL) will be collected by an experienced laboratory technician from the antecubital vein between 07:00 and 09:00 a.m. following an overnight fast (≥10 h). Participants will be instructed to refrain from vigorous physical activity for 24 h prior to each blood draw and to consume a standardized light meal on the evening before testing. Blood for plasma analysis will be collected into EDTA-containing tubes, whereas blood for serum analysis will be collected into serum separator tubes. All samples will be centrifuged within 10 min of collection at 4 °C and 3000 × g. Plasma and serum samples will then be stored at −80 °C until batch analysis. Plasma concentrations of GH, IGFBP-3, IL-6, CRP, and VEGF, as well as serum concentrations of testosterone and cortisol, will be determined using commercially available ELISA kits (ZellBio GmbH, Germany) in accordance with the manufacturers’ protocols. All measurements will be performed in a single analytical batch to minimize inter-assay variability (CV < 10%). Finally, the testosterone-to-cortisol ratio (T/C) will be calculated as serum testosterone concentration (nmol/L) divided by serum cortisol concentration (nmol/L).

##### 3.10.2 Secondary outcomes

Secondary outcomes will include cognitive function, physical performance, muscle strength, quality of life, and pain.

##### 3.10.2.1 Cognitive function

###### 3.10.2.1.1 Mini-mental state examination (MMSE)

The MMSE is a global cognitive screening tool that assesses five cognitive domains: orientation to time and place (10 points), registration (3 points), attention and calculation (5 points), recall (3 points), and language and praxis (9 points), yielding a total score ranging from 0 to 30 (34). Lower scores indicate greater cognitive impairment, with the commonly used clinical cut-off of ≤23 suggestive of dementia. The validated Persian version of the MMSE (30) will be administered by a trained assessor. In the Iranian validation study, the Persian MMSE demonstrated high test–retest reliability (r = 0.89, *p* < 0.001), as well as 100% sensitivity and 80% specificity at the cut-off score of 23 for detecting cognitive impairment in literate adults. In the present study, the MMSE will be used both at screening (a score of ≥24 will be required for study entry) and after the intervention to assess changes in global cognitive status.

###### 3.10.2.1.2 Montreal cognitive assessment (MoCA)

The MoCA is a more sensitive one-page 30-point screening instrument designed to detect mild cognitive impairment (MCI) and early dementia. It evaluates eight cognitive domains: visuospatial/executive functioning (5 points), naming (3 points), memory (5 points for delayed recall), attention (6 points), language (3 points), abstraction (2 points), and orientation (6 points). A score of ≥26 is generally considered normal, whereas scores below 26 indicate cognitive impairment. The MoCA has shown superior sensitivity compared with the MMSE for detecting MCI (90% vs. 18%) (35). The validated Persian version of the MoCA will be used in this study. The Persian MoCA has demonstrated satisfactory internal consistency (Cronbach's alpha = 0.76) and test–retest reliability (intraclass correlation coefficient [ICC] = 0.82), with a cut-off score of 23 providing optimal sensitivity (86%) and specificity (79%) for identifying MCI in Iranian older adults (36).

###### 3.10.2.1.3 Stroop test

The Stroop test is a classic neuropsychological measure of selective attention, cognitive inhibition, and processing speed. In the computerized version used in this study (CRSIB software), participants will be presented with colour words (red, blue, and green) displayed either in congruent ink colours or in incongruent ink colours. Participants will be instructed to identify the ink colour as quickly and accurately as possible while ignoring the semantic meaning of the word. The primary outcome will be the Stroop interference score, calculated as the mean reaction time (milliseconds) for correct incongruent trials minus the mean reaction time for correct congruent trials. Higher interference scores indicate poorer inhibitory control. The Stroop test has demonstrated excellent test–retest reliability in older adults (ICC = 0.86 for the interference score) and strong construct validity as a measure of executive control (37).

###### 3.10.2.1.4 Corsi block-tapping test

The Corsi block-tapping test is a visuospatial analogue of verbal memory tasks that assesses visuospatial short-term and working memory. In the computerized version used in this study (CRSIB software), nine identical blocks will be displayed on the screen in a fixed pseudo-random spatial arrangement. Participants will observe a sequence of highlighted blocks and will then be asked to reproduce the sequence either in the same order (forward condition, assessing short-term memory) or in reverse order (backward condition, assessing working memory). Sequence length will increase by one block after every two trials of the same length. The primary outcome will be the span length, defined as the longest sequence correctly reproduced. The Corsi test has shown acceptable test–retest reliability in older adults (ICC = 0.68–0.74) and good convergent validity with other measures of visuospatial memory (38).

##### 3.10.2.2. Physical and functional performance

###### 3.10.2.2.1 Muscle strength

Upper-body muscle strength will be assessed using a digital handgrip dynamometer. The participant will be seated with the shoulder adducted, the elbow flexed at 90°, and the forearm in a neutral position. Three maximal attempts will be performed with each hand, with 60 s of rest between attempts and alternating between hands. The highest value (kg) obtained across all six trials will be recorded. Handgrip dynamometry is a reliable (ICC = 0.97 in older women) and valid indicator of overall muscle strength and is predictive of functional decline and mortality (39). Lower-body muscle strength will be assessed using the leg press one-repetition maximum (1RM) test. Following a standardized warm-up, the estimated 1RM will be calculated using the validated Brzycki prediction equation, based on the maximum number of repetitions completed with a submaximal load. The mean of three test trials will be used for analysis. The leg press 1RM test has demonstrated excellent test–retest reliability (ICC = 0.99) in older adults (40).

###### 3.10.2.2.2 Functional performance

Functional mobility will be assessed using the Timed Up and Go (TUG) test. This test was selected as the primary functional outcome because it is widely used in older adults and reflects mobility, balance, transfer ability, and functional capacity related to fall risk. The participant will rise from a standard chair (seat height: 46 cm), walk 3 m at her usual pace, turn around a cone, return, and sit down again. Timing will begin on the command “go” and stop when the participant's back reaches the chair. The mean of three trials (seconds) will be used for analysis. The TUG has demonstrated excellent intra-rater (ICC = 0.99) and inter-rater reliability (ICC = 0.99) in community-dwelling older adults (41). A cut-off score of ≥13.5 s has been recommended to identify individuals at increased risk of falls in community settings (42).

Lower-limb functional muscle power will also be assessed using the Five-Repetition Sit-to-Stand (5×STS) test. This test was selected because it is widely used in older adults and reflects lower-extremity functional performance, transfer ability, and functional muscle power relevant to independence in daily activities. The participant will be seated on a standard chair without armrests, with arms crossed over the chest and feet placed flat on the floor. On the command “go,” she will be instructed to stand up fully and sit down five times as quickly as possible without using her arms. Timing will begin at the command “go” and stop when the participant reaches the full standing position at the end of the fifth repetition. One familiarization trial and two recorded trials will be performed, with adequate rest between trials, and the best time (seconds) will be used for analysis. The 5×STS test has demonstrated excellent test–retest reliability in older females (ICC = 0.95), with a standard error of measurement of 0.9 s and a minimal detectable change at the 95% confidence level of 2.5 s (43). In addition, a recent systematic review supported the reliability and clinical utility of the sit-to-stand tests in older adults, reinforcing their applicability for evaluating lower-extremity functional performance in this population (44).

##### 3.10.2.3 Quality of life and pain

Health-related quality of life will be assessed using the validated Iranian version of the 36-Item Short Form Health Survey (SF-36). The SF-36 is a generic self-report instrument comprising 36 items across eight multi-item subscales: physical functioning (10 items), role limitations due to physical health problems (4 items), bodily pain (2 items), general health perceptions (5 items), vitality (4 items), social functioning (2 items), role limitations due to emotional problems (3 items), and emotional well-being (5 items). One additional unscaled item assesses perceived change in health status over the past year. Scores for each subscale are transformed using a standardized scoring algorithm to a scale ranging from 0 (worst possible health) to 100 (best possible health). The Persian version of the SF-36 has been validated in the general Iranian population and has demonstrated satisfactory internal consistency across all subscales (Cronbach's alpha = 0.77–0.90, except for the vitality subscale, *α* = 0.65) and good construct validity, with factor analysis confirming a two-component summary structure representing physical and mental health, which explained 65.9% of the total variance (45).

##### 3.10.2.4 Pain intensity

Current pain intensity will be assessed using a linear Visual Analogue Scale (VAS) consisting of a 100-mm line anchored at the left end by “no pain” (score = 0) and at the right end by “worst imaginable pain” (score = 100). Participants will be asked to place a mark on the line corresponding to their current level of pain. The distance in millimetres from the left anchor to the participant's mark will constitute the pain score. The VAS has been widely validated in older populations and demonstrates strong test–retest reliability (ICC = 0.71–0.94) and construct validity, including convergent validity with the numerical rating scale (*r* = 0.80–0.95) (46). Higher VAS scores indicate greater pain intensity.

### 3.11 Statistical analysis

Data will be analyzed using IBM SPSS Statistics v25.0. Normality and homogeneity of variance will be assessed via Shapiro–Wilk and Levene's tests, respectively. Analysis of Covariance (ANCOVA) will be used to compare between-group differences post-intervention, using baseline values as covariates. Within-group changes will be assessed via paired t-tests. *Post-hoc* comparisons will utilize Bonferroni correction. Additionally, Pearson's correlation coefficient ® will be used to examine the strength and direction of linear relationships among the dependent variables.

Outcome analyses will include only participants who complete the study and have both baseline and post-intervention data for the outcome being analyzed (complete-case analysis). Participants who withdraw from the study will not be included in these analyses. Missing outcome data will not be imputed. The number of withdrawals and the reasons for withdrawal will be reported separately for each group.

Effect sizes will be reported as partial eta squared (*η*p2​), interpreted as small (0.01), medium (0.06), or large (0.14). Significance is set at *p* < 0.05.

#### 3.11.1 Data management

Each participant's information will be recorded using a unique identification code. Personal identifying information will be stored separately from the research data. Only authorized members of the research team will have access to identifiable information. Electronic files will be password-protected, and paper documents will be stored in a secure, locked location.

### 3.12. Post-trial care and dissemination policy

After completion of the study, any ongoing health concerns or adverse events will be managed by the study physician, and appropriate medical referrals will be arranged if necessary. Participants in the control group (CON) will be offered the opportunity to participate free of charge in the functional training programme without BFR after completion of the post-intervention assessments. The research team will remain available for one month after the end of the trial to address any questions or concerns raised by participants. The results of this study, regardless of the direction or magnitude of the findings, will be disseminated through publication in peer-reviewed scientific journals and presentation at national and international conferences. A plain-language summary of the key findings will be prepared and provided to all study participants.

## 4 Discussion

This study protocol describes a single-blind, three-arm randomized controlled trial designed to investigate the effects of an eight-week functional training program with blood flow restriction (FTBFR) on biochemical, cognitive, and functional outcomes, as well as quality of life and pain, in community-dwelling postmenopausal women. To our knowledge, few trials have simultaneously examined such a broad set of outcomes, including the GH/IGFBP-3 axis, testosterone-to-cortisol (T:C) ratio, IL-6, CRP, VEGF, cognitive function, upper- and lower-body muscle strength, functional performance, quality of life, and pain, within a single BFR-based functional training intervention specifically targeting postmenopausal women.

### 4.1 Rationale and scientific premise

Aging is associated with declines in anabolic hormonal activity, increased low-grade systemic inflammation, and impairments in physical and cognitive function. Evidence from systematic reviews and position statements indicates that low-load resistance training with BFR can improve muscle strength and hypertrophy while requiring substantially lower external loads than conventional resistance training, making it potentially suitable for Postmenopausal women and clinically vulnerable populations (17, 18, 32). Functional training, which emphasizes multi-joint movements related to activities of daily living, may improve mobility, balance, and functional independence in community-dwelling adults (21, 32, 47, 48). Combining functional training with BFR may therefore provide a practical low-load strategy to target multiple domains of healthy aging in Postmenopausal women.

The menopausal transition is associated with persistent estrogen deficiency, which may interact with age-related declines in anabolic hormonal activity, increased low-grade systemic inflammation, and impairments in physical and cognitive function. Evidence from systematic reviews and position statements indicates that low-load resistance training with BFR can improve muscle strength and hypertrophy while requiring substantially lower external loads than conventional resistance training, making it potentially suitable for postmenopausal women, particularly those who may not tolerate high-load exercise, and other clinically vulnerable populations (17, 20, 32). Functional training, which emphasizes multi-joint movements related to activities of daily living, may improve mobility, balance, and functional independence in postmenopausal and older adults (21, 32, 47, 48). Combining functional training with BFR may therefore provide a practical low-load strategy to target musculoskeletal, functional, and cognitive domains affected by menopause and aging in postmenopausal women.

### 4.2 GH/IGFBP-3 axis and VEGF-related adaptations

Previous mechanistic work has demonstrated that low-load resistance exercise with BFR can markedly increase acute GH responses and activate mTORC1 signaling and muscle protein synthesis in older men, despite the use of relatively light loads (20, 30, 48). Exercise interventions in older and postmenopausal women may also influence circulating growth-related markers, although hormonal responses appear to vary across studies (10, 21, 49). The present trial will therefore examine whether adding BFR to functional training is associated with favorable changes in GH and IGFBP-3 in community-dwelling postmenopausal women.

VEGF is also of interest because of its role in angiogenesis and tissue perfusion. Available evidence suggests that BFR may augment VEGF responses beyond those achieved with exercise alone, possibly through local hypoxic and vascular stimuli (14, 15, 17, 50). By assessing VEGF alongside cognitive and functional outcomes, this trial may help clarify whether angiogenic adaptations accompany broader neuromuscular and cognitive changes following FTBFR in postmenopausal women.

### 4.3 Anabolic-catabolic balance and the T:C ratio

BFR training may also influence the balance between anabolic and catabolic processes. Previous studies in older and postmenopausal populations have reported favorable changes in testosterone, cortisol, and the T:C ratio following low-load BFR exercise (9, 10). In addition, a recent meta-analysis suggested that low-load BFR training may induce greater improvements in markers related to muscle anabolism than low-load exercise alone or non-exercise conditions (51). These findings support the inclusion of the T:C ratio as a relevant outcome in the present trial and may be particularly relevant in the context of menopause-related hormonal alterations.

### 4.4 Inflammatory pathways

Chronic low-grade inflammation is a relevant feature of postmenopausal physiology and age-related functional decline. Previous trials and meta-analyses indicate that exercise training, including resistance-based interventions, may reduce inflammatory markers such as CRP and IL-6 in older adults (11, 14, 52). Because BFR enables training at lower mechanical loads, FTBFR may provide a tolerable approach for postmenopausal women while still eliciting beneficial anti-inflammatory adaptations.

### 4.5 Cognitive and functional outcomes

Exercise interventions that combine physical and cognitive demands have been associated with improvements in mobility, balance, muscle performance, and cognitive function in older adults (14, 53). Emerging evidence further suggests that BFR-based exercise may enhance some of these benefits, including cognitive and dual-task performance (17, 22). Given that functional training directly targets movement patterns relevant to daily life, the addition of BFR may offer a promising strategy to improve both functional independence and cognitive health in postmenopausal women, in whom estrogen deficiency may increase vulnerability to cognitive–motor decline.

### 4.6 Quality of life and pain

In postmenopausal women, patient-reported outcomes such as quality of life and pain are clinically meaningful. Multicomponent and resistance-based exercise programs have been associated with improvements in health-related quality of life and reductions in pain severity (20). Because FTBFR uses relatively low external loads, it may represent a feasible option for postmenopausal women who are reluctant or unable to engage in higher-load exercise.

### 4.7 Knowledge gaps and trial rationale

Despite growing interest in BFR exercise, important gaps remain. Many previous studies have included men or mixed-sex samples, and relatively few trials have focused exclusively on postmenopausal women. In addition, few studies have simultaneously assessed a broad range of biochemical, cognitive, and functional outcomes within a single functional training plus BFR intervention. The present trial is designed to address these gaps by evaluating GH, IGFBP-3, the T:C ratio, IL-6, CRP, VEGF, cognitive performance, muscle strength, mobility, quality of life, and pain after an eight-week FTBFR program in community-dwelling postmenopausal women.

### 4.8 Strengths

A major strength of this trial is its integrated, multi-domain assessment. The study will evaluate biomarkers related to anabolic activity (GH, IGFBP-3, testosterone), catabolic status (cortisol and the T:C ratio), inflammation (IL-6, CRP), and angiogenesis (VEGF), alongside cognitive performance (MMSE, MoCA, Stroop, and Corsi tests) and functional outcomes, including handgrip strength, lower-body strength, and mobility. Another strength is the exclusive focus on community-dwelling postmenopausal women, a population that remains underrepresented in BFR research despite experiencing menopause-specific hormonal, musculoskeletal, vascular, and cognitive changes.

### 4.9 Limitations

Several limitations should be acknowledged. First, the single-center design and the inclusion of generally healthy, community-dwelling postmenopausal women may limit generalizability to frailer populations, women with substantial comorbidity, or those in different stages of the menopausal transition. Second, the eight-week intervention does not allow assessment of long-term maintenance or clinical outcomes such as falls. Third, although outcome assessors will be blinded to group allocation, participants cannot be blinded to exercise participation, which may introduce expectancy effects. Fourth, ongoing heterogeneity in cuff characteristics, occlusion pressures, and progression strategies across BFR studies may complicate comparison with the wider literature (17–19). Finally, estimating arterial occlusion pressure using anthropometric-based formulas rather than Doppler ultrasound may introduce variability in the individualized BFR stimulus.

### 4.10 Future directions

Future studies should include larger and more diverse samples of postmenopausal women, longer follow-up periods, and greater methodological standardization of BFR application, including individualized Doppler-based occlusion assessment where feasible. Additional work is also needed to examine long-term safety and to explore mechanisms underlying potential cognitive benefits, for example through neuroimaging or biomarkers of neuroplasticity such as BDNF. Studies comparing women at different stages of the menopausal transition and postmenopause may also help clarify whether the timing of estrogen loss modifies the response to FTBFR.

## 5 Conclusion

If effective, FTBFR may represent a practical, low-load, and time-efficient exercise strategy to support muscle health, functional independence, and cognitive well-being in postmenopausal women. Given the potential contribution of estrogen deficiency to declines in physical and cognitive function, interventions that can be implemented in structured exercise settings are of clear clinical and public health relevance. The findings of this trial may help inform future exercise prescription and provide preliminary evidence for integrating BFR into functional training programs specifically designed for postmenopausal women.

## References

[1] Masini A Cherasco N Conti A Pighini I Barone-Adesi F Panella M. Preventive pathways for healthy ageing: a systematic literature review. Geriatrics. (2025) 10(1):31. 10.3390/geriatrics10010031 39997530 PMC11855837

[2] Moscucci F Baratta F Bucciarelli V Mattioli AV Pinti M Zanini G. Inflammaging and cardiovascular risk in old women. High Blood Press Cardiovasc Prev. (2026) 33(2):171–81. 10.1007/S40292-025-00758-1 41366614 PMC12989026

[3] Beaudart C Alcazar J Aprahamian I Batsis JA Yamada Y Prado CM. Health outcomes of sarcopenia: a consensus report by the outcome working group of the global leadership initiative in sarcopenia (GLIS). Aging Clin Exp Res. (2025) 37(1):100. 10.1007/s40520-025-02995-9 40120052 PMC11929733

[4] Wright VJ Schwartzman JD Itinoche R Wittstein J. The musculoskeletal syndrome of menopause. Climacteric. (2024) 27(5):466–72. 10.1080/13697137.2024.2380363 39077777

[5] Tu S Hao X Xu S Jin X Liao W Xia H. Sarcopenia: current insights into molecular mechanisms, diagnostics, and emerging interventional approaches. Int J Mol Sci. (2025) 26(14):6740. 10.3390/ijms26146740 40724988 PMC12295260

[6] Damanti S Senini E De Lorenzo R Merolla A Santoro S Festorazzi C. Molecular constraints of sarcopenia in the ageing muscle. Frontiers in Aging. (2025) 6:1588014. 10.3389/fragi.2025.1588014 40678078 PMC12267276

[7] Xu M Liu X. Ribosome biogenesis and translational control in skeletal muscle atrophy and hypertrophy: mechanisms and therapeutic perspectives. Biomolecules. (2026) 16(3):406. 10.3390/biom16030406 41897342 PMC13023859

[8] Witkowska-Sędek E Pyrżak B. Chronic inflammation and the growth hormone/insulin-like growth factor-1 axis. Central European Journal of Immunology. (2020) 45(4):469–75. 10.5114/ceji.2020.103422 PMC7882400 33613096

[9] Zouraghi MR Barjeste Yazdi A Khajei R Rashidlamir A. The effect of A period of resistance training and blood flow restriction on body fat percentage, muscle mass and testosterone to cortisol ratio in the elderly. Razi J Med Sci. (2023) 30:105. 10.47176/rjms.30.103

[10] Farhadiyani asgarabadi M Behpoor NM Moghaddam A. Changes in some fitness factors, testosterone, and cortisol following combined exercise with and without blood flow restriction in older men. yafte. (2022) 24(1):58–74. 10.32592/Yafteh.2022.24.1.6

[11] Denson LA Held MA Menon RK Frank SJ Parlow AF Arnold DL. Interleukin-6 inhibits hepatic growth hormone signaling via upregulation of cis and socs-3. American Journal of Physiology-Gastrointestinal and Liver Physiology. (2003) 284(4):G646–54. 10.1152/ajpgi.00178.2002 12519742

[12] Ownby RL. Neuroinflammation and cognitive aging. Curr Psychiatry Rep. (2010) 12(1):39–45. 10.1007/s11920-009-0082-1 20425309

[13] Jin R Chan AK Wu J Lee TM. Relationships between inflammation and age-related neurocognitive changes. Int J Mol Sci. (2022) 23(20):12573. 10.3390/ijms232012573 36293430 PMC9604276

[14] Tangestani Fard M Stough C. A review and hypothesized model of the mechanisms that underpin the relationship between inflammation and cognition in the elderly. Front Aging Neurosci. (2019) 11:56. 10.3389/fnagi.2019.00056 30930767 PMC6425084

[15] Agrawal N Afzal M Khan NH Ganesan S Kumari M Sunitha S. The role of VEGF in vascular dementia: impact of aging and cellular senescence. Biogerontology. (2025) 26(2):77. 10.1007/s10522-025-10219-w 40119956

[16] Mekala A Qiu H. Interplay between vascular dysfunction and neurodegenerative pathology: new insights into molecular mechanisms and management. Biomolecules. (2025) 15(5):712. 10.3390/biom15050712 40427605 PMC12109301

[17] Kargaran A Abedinpour A Saadatmehr Z Yaali R Amani-Shalamzari S Gahreman D. Effects of dual-task training with blood flow restriction on cognitive functions, muscle quality, and circulatory biomarkers in elderly women. Physiol Behav. (2021) 239:113500. 10.1016/j.physbeh.2021.113500 34174326

[18] Emam MA Elsayed A Hortobágyi T Amin WM Malik S Ali OI. Exploring blood flow restriction exercise protocols for elderly populations: a scoping review of cuff pressure, frequency, and duration for muscle strength, hypertrophy, and functional abilities outcomes. J Clin Med. (2025) 14(12):4185. 10.3390/jcm14124185 40565940 PMC12193843

[19] Pandit A Tran TB Letton M Cowley E Gibbs M Wewege MA. Data informing governing body resistance-training guidelines exhibit sex bias: an audit-based review. Sports Med. (2023) 53(9):1681–91. 10.1007/s40279-023-01878-1 37382828 PMC10432356

[20] Feng M Li J Zhao J Pan X Wang M Han Q. Effect of blood flow restriction training on health promotion in middle-aged and elderly women: a systematic review and meta-analysis. Front Physiol. (2024) 15:1392483. 10.3389/fphys.2024.1392483 39015223 PMC11249765

[21] Liu CJ Chang WP Shin YC Hu YL Morgan-Daniel J. Is functional training functional? A systematic review of its effects in community-dwelling older adults. Eur Rev Aging Phys Act. (2024) 21(1):32. 10.1186/s11556-024-00366-3 39716049 PMC11664925

[22] Chen YC Lo IP Tsai YY Zhao CG Hwang IS. Dual-task improvement of older adults after treadmill walking combined with blood flow restriction of low occlusion pressure: the effect on the heart–brain axis. J Neuroeng Rehabil. (2024) 21(1):116. 10.1186/s12984-024-01412-y 38997727 PMC11241870

[23] Hopewell S Chan A-W Collins GS Hróbjartsson A Moher D Schulz KF. CONSORT 2025 statement: updated guideline for reporting randomised trials. Lancet. (2025) 405(10489):1633–40. 10.1001/jama.2025.4347 40245901

[24] Hoffmann TC Glasziou PP Boutron I Milne R Perera R Moher D. Better reporting of interventions: template for intervention description and replication (TIDieR) checklist and guide. Br Med J. (2014) 7:348. 10.1136/bmj.g1687 24609605

[25] Lee PH Macfarlane DJ Lam TH Stewart SM. Validity of the international physical activity questionnaire short form (IPAQ-SF): a systematic review. Int J Behav Nutr Phys Act. (2011) 8(1):115. 10.1186/1479-5868-8-115 22018588 PMC3214824

[26] Herr KA Spratt K Mobily PR Richardson G. Pain intensity assessment in older adults: use of experimental pain to compare psychometric properties and usability of selected pain scales with younger adults. Clin J Pain. (2004) 20(4):207–19. 10.1097/00002508-200407000-00002 15218405

[27] Rashedi V Foroughan M Chehrehnegar N. Psychometric properties of the Persian Montreal cognitive assessment in mild cognitive impairment and Alzheimer disease. Dement Geriatr Cogn Dis Extra. (2021) 11(1):51–7. 10.1159/000514673 33976692 PMC8077519

[28] Faul F Erdfelder E Lang AG Buchner A. G* power 3: a flexible statistical power analysis program for the social, behavioral, and biomedical sciences. Behav Res Methods. (2007) 39(2):175–91. 10.3758/BF03193146 17695343

[29] Cohen J. Statistical Power Analysis for the Behavioral Sciences. New York, NY: Routledge (2013). 10.4324/9780203771587

[30] Aram S Khodaei K Zolfaghar Didani M. The effect of low-intensity suspension training with blood flow restriction on GH, IGF-1, and their association with physical fitness in young women. Physiol Rep. (2024) 12(15):e16154. 10.14814/phy2.16154 39095334 PMC11296940

[31] Knutzen KM Brilla LR Caine D. Validity of 1RM prediction equations for older adults. The Journal of Strength & Conditioning Research. (1999) 13(3):242–6. 10.1519/00124278-199908000-00011

[32] Pazokian F Amani-Shalamzari S Rajabi H. Effects of functional training with blood occlusion on the irisin, follistatin, and myostatin myokines in elderly men. Eur Rev Aging Phys Act. (2022) 19(1):22. 10.1186/s11556-022-00303-2 36153484 PMC9508759

[33] Patterson SD Hughes L Warmington S Burr J Scott BR Owens J. Blood flow restriction exercise: considerations of methodology, application, and safety. Front Physiol. (2019) 10:533. 10.3389/fphys.2019.00533 31156448 PMC6530612

[34] Tombaugh TN McIntyre NJ. The mini-mental state examination: a comprehensive review. J Am Geriatr Soc. (1992) 40(9):922–35. 10.1111/j.1532-5415.1992.tb01992.x 1512391

[35] Seyedian M Fallah M Norouzian M Nejat S Delavar A Ghasemzadeh H. Validity of the farsi version of mini-mental state examination. J Med Counc Islam Repub Iran. (2007) 25(4):408–14.

[36] Nasreddine ZS Phillips NA Bédirian V Charbonneau S Whitehead V Collin I. The Montreal cognitive assessment, MoCA: a brief screening tool for mild cognitive impairment. J Am Geriatr Soc. (2005) 53(4):695–9. 10.1111/j.1532-5415.2005.53221.x 15817019

[37] Scarpina F Tagini S. The stroop color and word test. Front Psychol. (2017) 8:241674. 10.3389/fpsyg.2017.00557 PMC5388755 28446889

[38] Kessels RP Van Zandvoort MJ Postma A Kappelle LJ De Haan EH. The corsi block-tapping task: standardization and normative data. Appl Neuropsychol. (2000) 7(4):252–8. 10.1207/S15324826AN0704_8 11296689

[39] Roberts HC Denison HJ Martin HJ Patel HP Syddall H Cooper C. A review of the measurement of grip strength in clinical and epidemiological studies: towards a standardised approach. Age Ageing. (2011) 40(4):423–9. 10.1093/ageing/afr051 21624928

[40] Levinger I Goodman C Hare DL Jerums G Toia D Selig S. The reliability of the 1RM strength test for untrained middle-aged individuals. Journal of Science and Medicine in Sport. (2009) 12(2):310–6. 10.1016/j.jsams.2007.10.007 18078784

[41] Podsiadlo D Richardson S. The timed “up & go”: a test of basic functional mobility for frail elderly persons. J Am Geriatr Soc. (1991) 39(2):142–8. 10.1111/j.1532-5415.1991.tb01616.x 1991946

[42] Sadeghi H Jehu DA Daneshjoo A Shakoor E Razeghi M Amani A. Effects of 8 weeks of balance training, virtual reality training, and combined exercise on lower limb muscle strength, balance, and functional mobility among older men: a randomized controlled trial. Sports Health. (2021) 13(6):606–12. 10.1177/1941738120986803 33583253 PMC8558995

[43] Goldberg A Chavis M Watkins J Wilson T. The five-times-sit-to-stand test: validity, reliability and detectable change in older females. Aging Clin Exp Res. (2012) 24(4):339–44. 10.1007/BF03325265 23238309

[44] Muñoz-Bermejo L Adsuar JC Mendoza-Muñoz M Barrios-Fernández S Garcia-Gordillo MA Pérez-Gómez J. Test-retest reliability of five times sit to stand test (FTSST) in adults: a systematic review and meta-analysis. Biology (Basel). (2021) 10(6):510. 10.3390/biology10060510 PMC8228261 34207604

[45] Montazeri A Goshtasebi A Vahdaninia M Gandek B. The short form health survey (SF-36): translation and validation study of the Iranian version. Qual Life Res. (2005) 14(3):875–82. 10.1007/s11136-004-1014-5 16022079

[46] Mehdizadeh M Fereshtehnejad SM Goudarzi S Moshtagh A Dehghanian Nasrabadi F Habibi SA. Validity and reliability of short-form McGill pain questionnaire-2 (SF-MPQ-2) in Iranian people with Parkinson’s disease. Parkinson’s Disease. (2020) 2020(1):2793945. 10.1155/2020/2793945 PMC7450331 32908666

[47] Hu X Li Y Jiang H Luo Z Zengqing F Zou J. Baduanjin exercise improves functional capacity and cardiovascular function in older adults with cardiovascular diseases: a systematic review and meta-analysis. Front Cardiovasc Med. (2025) 12:1419095. 10.3389/fcvm.2025.1419095 40342973 PMC12058754

[48] Fry CS Glynn EL Drummond MJ Timmerman KL Fujita S Abe T. Blood flow restriction exercise stimulates mTORC1 signaling and muscle protein synthesis in older men. J Appl Physiol. (2010) 108(5):1199–209. 10.1152/japplphysiol.01266.2009 20150565 PMC2867530

[49] Bigdeli S Dehghaniyan MH Amani-Shalamzari S Rajabi H Gahreman DE. Functional training with blood occlusion influences muscle quality indices in older adults. Arch Gerontol Geriatr. (2020) 90:104110. 10.1016/j.archger.2020.104110 32473556

[50] Larkin KA Macneil RG Dirain M Sandesara B Manini TM Buford TW. Blood flow restriction enhances post–resistance exercise angiogenic gene expression. Med Sci Sport Exerc. (2012) 44:2077–83. 10.1249/MSS.0b013e3182625928 PMC3633075 22677927

[51] Taylor CW Ingham SA Ferguson RA. Acute and chronic effect of sprint interval training combined with postexercise blood-flow restriction in trained individuals. Exp Physiol. (2016) 101(1):143–54. 10.1113/EP085293 26391312

[52] Shimizu R Hotta K Yamamoto S Matsumoto T Kamiya K Kato M. Low-intensity resistance training with blood flow restriction improves vascular endothelial function and peripheral blood circulation in healthy elderly people. Eur J Appl Physiol. (2016) 116(4):749–57. 10.1007/s00421-016-3328-8 26822582

[53] Fabero-Garrido R Gragera-Vela M Del Corral T Hernández-Martín M Plaza-Manzano G López-de-Uralde-Villanueva I. López-de-uralde-villanueva I. Effects of low-load blood flow restriction training on muscle anabolism biomarkers and thrombotic biomarkers compared with traditional training in healthy adults older than 60 years: systematic review and meta-analysis. Life. (2024) 14(3):411. 10.3390/life14030411 38541735 PMC10971244

