# Supplementary material for: Effects of eight weeks of functional training with blood flow restriction on biochemical, cognitive, and functional outcomes in postmenopausal women: protocol for a single-blind randomized controlled trial
Source: Front Sports Act Living. 2026 Oct 27;8:1953943. doi: 10.3389/fspor.2026.1953943 (PMC13748257; doi:10.3389/fspor.2026.1953943)
Supplement: Supplementary file 1 [file Table1.docx]

**CONSORT 2025 checklist of information to include when reporting a randomised trial***

| **Section / Topic** | **No** | **CONSORT 2025 checklist item description** | **Reported on page no.** |
| --- | --- | --- | --- |
| **Title and abstract** | | |  |
| Title and structured abstract | 1a | Effects of Eight Weeks of Functional Training with Blood Flow Restriction on Biochemical, Cognitive, And Functional Outcomes in Postmenopausal Women: Protocol for a Single-Blind Randomized Controlled Trial |  |
|  | 1b | Introduction: Aging in women is accompanied by impaired muscle and cognitive function, reduced anabolic activity, and chronic low-grade inflammation. Blood flow restriction (BFR) may enhance adaptations to low-load exercise, but its effects when combined with functional training in postmenopausal women are unclear. This trial will examine the effects of eight weeks of functional training with BFR on biochemical, cognitive, and functional outcomes in postmenopausal women. Methods: This protocol describes a single-blind, assessor-masked, randomized controlled trial that will recruit 45 eligible postmenopausal women, defined as having experienced no menstrual bleeding for at least 24 consecutive months before screening, without another identifiable physiological or pathological cause. After health screening and physician-confirmed examination, participants will be randomly assigned (1:1:1) to functional training with BFR (FTBFR; n=15), functional training alone (FT; n=15), or non-exercise control (CON; n=15) using web-based randomization. Exercise groups will complete supervised sessions three times weekly for eight weeks. Primary outcomes are plasma/serum concentrations of growth hormone, insulin-like growth factor-binding protein-3, interleukin-6, C-reactive protein, vascular endothelial growth factor, testosterone, and cortisol, measured by ELISA; the testosterone-to-cortisol ratio will also be calculated. Cognitive outcomes include the Mini-Mental State Examination, Montreal Cognitive Assessment, Stroop test, and Corsi block-tapping test. Secondary outcomes are handgrip strength, upper- and lower-body muscle strength, Timed Up and Go five-times-sit-to-stand performance, quality of life (SF-36), and pain. Conclusions: This trial will clarify whether adding BFR to functional training provides additional biochemical, cognitive, and functional benefits in Postmenopausal women and may inform safe, low-intensity exercise strategies for healthy aging. |  |
| **Open science** | | |  |
| Trial registration | 2 | The trial was prospectively registered in the Iranian Registry of Clinical Trials (IRCT) under registration number IRCT20191123045478N4; on 25 May 2026. |  |
| Protocol and statistical analysis plan | 3 | The full trial protocol and statistical analysis plan will be available from the corresponding author upon reasonable request. Important protocol amendments will also be updated in the trial registry and documented in future publications. |  |
| Data sharing | 4 | De-identified individual participant data, study protocol, statistical code, and data dictionary will be available from the corresponding author upon reasonable request following publication of the primary study findings.  Data access may require approval by the research team and compliance with institutional confidentiality regulations. |  |
| Funding and conflicts of interest | 5a | This study will be supported by Kharazmi University, and the publication costs will be covered by the Research Office of the United Arab Emirates University. |  |
|  | 5b | The authors declare no financial or non-financial conflicts of interest related to this study. |  |
| **Introduction** | | |  |
| Background and rationale | 6 | Menopause is associated with progressive declines in muscle strength, functional mobility, cognitive performance, and anabolic hormonal activity, together with increased systemic inflammation. These changes may contribute to reduced physical independence, impaired quality of life, and an increased risk of functional disability among postmenopausal women.  Functional training has been shown to improve mobility, balance, and muscular performance in postmenopausal women and older adults. Blood flow restriction (BFR) training is a low-load exercise strategy that may induce muscular and hormonal adaptations through localized hypoxia and metabolic stress while minimizing mechanical loading.  Previous studies have demonstrated beneficial effects of both functional training and BFR on muscle strength and functional performance. However, limited evidence is available regarding the combined effects of these approaches on biochemical, cognitive, and functional outcomes in postmenopausal women.  Therefore, this randomized controlled trial is designed to evaluate whether functional training combined with BFR produces superior adaptations compared with functional training alone and a non-exercise control condition in postmenopausal women. |  |
| Objectives | 7 | The primary objective of this randomized controlled trial is to determine the effects of eight weeks of functional training combined with blood flow restriction on biochemical markers, including GH, IGFBP-3, IL-6, CRP, VEGF, and the testosterone-to-cortisol ratio (TCR), in postmenopausal women. Secondary objectives are to evaluate changes in cognitive performance, muscle strength, functional mobility, pain intensity, and quality of life.  The study also aims to assess the safety, tolerability, and adherence associated with low-load blood flow restriction exercise in postmenopausal women.  We hypothesize that functional training combined with BFR will result in greater improvements in anabolic, inflammatory, cognitive, and functional outcomes compared with functional training alone and a non-exercise control group. |  |
| **Methods** | | |  |
| Patient and public involvement | 8 | Patients and members of the public will not be formally involved in the design, conduct, reporting, or dissemination plans of this randomized controlled trial.  However, preliminary feedback obtained during familiarization sessions with postmenopausal women will be considered to improve the feasibility, safety, and acceptability of the exercise intervention. |  |
| Trial design | 9 | This study is designed as a single-center, single-blind, three-arm parallel-group randomized controlled superiority trial with a 1:1:1 allocation ratio.  Participants will be randomly assigned to one of three groups:  (1) functional training with blood flow restriction (FTBFR),  (2) functional training alone (FT), or  (3) a non-exercise control group (CON).  The intervention period will last eight weeks, with supervised exercise sessions conducted three times weekly. |  |
| Changes to trial protocol | 10 | At the time of manuscript submission, no important modifications to the trial protocol, outcomes, or statistical analyses have occurred.  Any future protocol amendments will be approved by the institutional ethics committee and updated in the trial registry. |  |
| Trial setting | 11 | The trial will be conducted in Tehran, Iran.  Participant recruitment will take place through advertisements and announcements distributed at community centers, healthcare facilities, and sports clubs.  Baseline and post-intervention assessments will be performed in the Exercise Physiology Research Laboratory at Kharazmi University, and all exercise sessions will be supervised at Barman Sports Club. |  |
| Eligibility criteria | 12a | Eligible participants will be community-dwelling postmenopausal women who have experienced no menstrual bleeding for at least 24 consecutive months before screening, in the absence of any other identifiable physiological or pathological cause, lead a sedentary or low-active lifestyle, and have been medically cleared to participate in exercise training involving blood flow restriction (BFR).Participants must have an MMSE score ≥24 and no history of severe cardiovascular, neurological, musculoskeletal, or metabolic disorders.  Exclusion criteria will include participation in structured exercise programs during the previous six months, initiation of medications or supplements affecting study outcomes, contraindications to BFR exercise, poor intervention adherence, or occurrence of adverse events requiring discontinuation. |  |
|  | 12b | Exercise sessions will be supervised by qualified exercise physiologists experienced in functional training and blood flow restriction exercise prescription.  Outcome assessors will receive standardized training before data collection to ensure consistency of testing procedures. |  |
| Intervention and comparator | 13 | Participants allocated to the FTBFR and FT groups will complete an 8-week supervised exercise program consisting of three sessions per week on non-consecutive days.  Each session will include warm-up, circuit-based functional exercises, and cool-down periods. Functional exercises will target upper- and lower-body muscle groups using dumbbells, medicine balls, Swiss balls, and BOSU balls, with progressive increases in exercise intensity throughout the intervention.  In the FTBFR group, pneumatic cuffs will be applied to the proximal upper arms and thighs during exercise sessions. Blood flow restriction pressure will be prescribed as a percentage of estimated arterial occlusion pressure (AOP) and progressively increased throughout the study.  Participants in the FT group will perform the identical exercise protocol without blood flow restriction. Participants assigned to the control group will maintain their usual daily activities and will not participate in structured exercise training during the study period.  Detailed intervention procedures and progression protocols are provided in the supplementary intervention tables. |  |
| Outcomes | 14 | Primary outcomes will include serum biochemical markers, including GH, IGFBP-3, IL-6, CRP, VEGF, and the testosterone-to-cortisol ratio (TCR). Secondary outcomes will include cognitive performance assessed by the Montreal Cognitive Assessment (MoCA), Mini-Mental State Examination (MMSE), Stroop test, and Corsi Block-Tapping Test; muscle strength assessed by handgrip strength and one-repetition maximum (1RM) leg press strength; functional mobility assessed using the Timed Up and Go (TUG) and Go five-times-sit-to-stand test; pain intensity assessed by the Visual Analog Scale (VAS); and quality of life assessed using the SF-36 questionnaire.  All outcomes will be assessed at baseline and 48 hours after completion of the 8-week intervention period.  Continuous variables will be analyzed as changes from baseline to post-intervention and summarized using means and standard deviations. |  |
| Harms | 15 | Adverse events and harms will be assessed systematically throughout the intervention period during all supervised exercise sessions.  Particular attention will be given to blood flow restriction-related complications, including excessive pain, dizziness, numbness, bruising, paresthesia, exercise intolerance, and symptoms suggestive of thrombotic complications.  All adverse events will be documented and evaluated by the supervising research team. |  |
| Sample size | 16a | The sample size was calculated a priori using G*Power version 3.1.9.6 (Heinrich Heine University Düsseldorf, Düsseldorf, Germany) (29). The calculation was based on a repeated-measures ANCOVA framework with three groups (FTBFR, FT, and CON) and two assessment points (baseline and post-intervention). With a two-sided α of 0.05, power of 80%, an assumed medium effect size of f = 0.25 (30,31), and a within-subject correlation of 0.70, the minimum required sample size was 36 participants (12 per group). Allowing for an anticipated attrition rate of approximately 15%, the target enrolment was increased to 45 participants (15 per group). Participant recruitment, allocation, and follow-up will be presented in the trial flow diagram |  |
|  | 16b | No formal interim analyses or stopping guidelines are planned because of the relatively small sample size, short intervention duration, and low-risk nature of the exercise intervention. |  |
| Randomisation: |  |  |  |
| Sequence generation | 17a | The random allocation sequence will be generated by an independent researcher using a computer-generated randomization procedure created with Random Allocation Software. |  |
|  | 17b | Participants will be randomized using restricted block randomization with a 1:1:1 allocation ratio.  Variable block sizes will be used to maintain balanced allocation and reduce predictability of group assignment.  No stratification procedures will be applied. |  |
| Allocation concealment mechanism | 18 | The allocation sequence will be implemented using sequentially numbered, opaque, sealed envelopes prepared by an independent researcher not involved in participant recruitment, assessment, or intervention delivery.  Envelopes will be opened sequentially only after completion of baseline assessments. |  |
| Implementation | 19 | Participant eligibility assessment and enrollment will be conducted by members of the research team who will not have access to the random allocation sequence.  Group assignment will be performed after baseline assessments using the concealed allocation envelopes prepared by the independent researcher. |  |
| Blinding | 20a | This study will be conducted as a single-blind randomized controlled trial.  Outcome assessors and data analysts will be blinded to participant allocation throughout the study.  Because of the nature of the intervention, participants and exercise supervisors cannot be blinded. |  |
|  | 20b | Outcome assessments will be conducted by researchers who are not involved in intervention delivery or participant allocation.  Participants will be instructed not to disclose their group assignment during assessments.  Data analyses will be performed using coded group identifiers.  The FTBFR and FT interventions will be structurally similar in exercise frequency, duration, supervision, and training environment, differing only in the application of blood flow restriction cuffs. |  |
| Statistical methods | 21a | Statistical analyses will be performed using IBM SPSS Statistics software (version 27.0).  Continuous variables will be expressed as mean ± standard deviation (SD).  Baseline normality assumptions will be assessed using the Shapiro–Wilk test, and homogeneity of variances will be evaluated using Levene’s test.  Between-group differences in primary and secondary outcomes will be analyzed using analysis of covariance (ANCOVA), with baseline values entered as covariates.  Bonferroni-adjusted post hoc tests will be used when significant overall group effects are detected.  Effect sizes will be calculated using partial eta squared (η²p), and statistical significance will be established at p < 0.05.  Harms and adverse events will be summarized descriptively. |  |
|  | 21b | Primary analyses will follow the intention-to-treat principle and will include all randomized participants according to their originally assigned groups.  Secondary per-protocol analyses may additionally be performed, including participants who complete at least 85% of scheduled intervention sessions. |  |
|  | 21c | Missing outcome data will be evaluated for patterns of missingness and handled using appropriate imputation procedures when necessary.  Sensitivity analyses comparing complete-case and imputed datasets may also be conducted. |  |
|  | 21d | Prespecified additional analyses will include sensitivity analyses comparing intention-to-treat and per-protocol findings.  Exploratory analyses examining associations between biochemical, cognitive, and functional adaptations will also be planned.  No formal subgroup analyses are prespecified because of the relatively small sample size. |  |
| **Results** | | |  |
| Participant flow, including flow diagram | 22a | Participant flow through the trial will be reported in accordance with the CONSORT guidelines using a standardized flow diagram.  The diagram will present the numbers of participants:   - screened for eligibility, - excluded (with reasons), - randomly assigned, - allocated to each study group, - receiving the intended intervention, - completing follow-up assessments, and - included in the primary outcome analyses. |  |
|  | 22b | Losses to follow-up, withdrawals, exclusions after randomization, and reasons for discontinuation will be documented and reported separately for each study group in the final trial report. |  |
| Recruitment | 23a | Participant recruitment is planned to commence on 15 June 2026 and is expected to continue until 21 August 2026. Outcome assessments will be conducted at baseline and 48 hours after completion of the 8-week intervention period. |  |
|  | 23b | At the time of protocol submission, the trial has not been stopped or terminated.  Premature termination may occur in the event of serious safety concerns, insufficient recruitment, or institutional decisions affecting trial feasibility. |  |
| Intervention and comparator delivery | 24a | Exercise sessions will be supervised by qualified exercise physiologists to ensure standardized intervention delivery and protocol fidelity.  Attendance, exercise intensity, blood flow restriction pressure, and participant adherence will be monitored and documented throughout the intervention period.  Intervention fidelity will be evaluated using session attendance records, exercise logs, and supervision checklists. |  |
|  | 24b | Participants will be instructed to maintain their usual dietary habits and daily physical activity levels throughout the study period.  Participation in additional structured exercise programs, resistance training, or initiation of medications or supplements affecting study outcomes will be prohibited during the intervention period.  Any concomitant treatments or changes in medication status will be documented during follow-up. |  |
| Baseline data | 25 | Baseline demographic and clinical characteristics will be summarized for each study group using descriptive statistics in the final trial report.  Variables will include age, anthropometric characteristics, baseline biochemical measures, cognitive performance scores, and functional outcomes. |  |
| Numbers analysed,  outcomes and estimation | 26 | - Final analyses will report, for each study group: - the number of randomized participants, - the number included in each analysis, - the number with available outcome data, - estimated intervention effects, - corresponding effect sizes, and - 95% confidence intervals. - Continuous outcomes will be analyzed as changes from baseline to post-intervention using ANCOVA adjusted for baseline values. - Results for both primary and secondary outcomes will be presented in accordance with CONSORT reporting recommendations. |  |
| Harms | 27 | All adverse events, unintended effects, and exercise-related complications occurring during the intervention period will be documented and reported separately for each study group in the final trial publication.  Particular attention will be given to adverse effects potentially associated with blood flow restriction exercise. |  |
| Ancillary analyses | 28 | Prespecified ancillary analyses will include sensitivity analyses comparing intention-to-treat and per-protocol findings.  Exploratory analyses examining relationships between biochemical, cognitive, and functional adaptations may also be conducted.  Any post-hoc analyses performed after completion of the trial will be clearly identified as exploratory in the final report. |  |
| **Discussion** | | |  |
| Interpretation | 29 | The findings of this trial are expected to improve understanding of the physiological, cognitive, and functional effects of combining functional training with blood flow restriction in postmenopausal women.  The study may provide evidence regarding the feasibility, safety, and potential clinical applicability of low-load blood flow restriction exercise in aging populations |  |
| Limitations | 30 | Potential limitations of the study may include the relatively small sample size, single-center design, inability to blind participants and exercise supervisors, and limited generalizability to other populations.  Additionally, the relatively short intervention duration may limit evaluation of long-term adaptations and sustainability of intervention effects.  Despite these limitations, the randomized controlled design, assessor blinding, and supervised intervention procedures are expected to strengthen the internal validity of the trial. |  |

*We strongly recommend reading this statement in conjunction with the CONSORT 2025 Explanation and Elaboration and/or the CONSORT 2025 Expanded Checklist for important clarifications on all the items. We also recommend reading relevant CONSORT extensions. See [www.consort-spirit.org](http://www.consort-spirit.org).

Citation: Hopewell S, Chan AW, Collins GS, Hróbjartsson A, Moher D, Schulz KF, et al. CONSORT 2025 Statement: updated guideline for reporting randomised trials. BMJ. 2025; 388:e081123. <https://dx.doi.org/10.1136/bmj-2024-081123>.

© 2025 Hopewell et al. This is an Open Access article distributed under the terms of the Creative Commons Attribution License (<https://creativecommons.org/licenses/by/4.0/>), which permits unrestricted use, distribution, and reproduction in any medium, provided the original work is properly cited.
